# Effect of a grace period on false alarm rates of smartwatch-based out-of-hospital cardiac arrest detection systems: a pilot study

**DOI:** 10.1016/j.resplu.2025.101215

**Published:** 2026-01-05

**Authors:** Roelof G. Hup, Chaimae Bouchnaf, Myrthe A. Plaisier, Fatuma M.A. Omar, Tobias A. Machiavello, Sophie L.M. van Spreuwel, Hanno L. Tan, Xi Long, Rik Vullings

**Affiliations:** a Department of Electrical Engineering, Eindhoven University of Technology, Eindhoven, the Netherlands; b Department of Experimental Cardiology, Heart Center, Amsterdam University Medical Center, University of Amsterdam, the Netherlands; c Human Technology Interaction Group, Eindhoven University of Technology, Eindhoven, the Netherlands; d Department of Mathematics and Computer Science, Eindhoven University of Technology, Eindhoven, the Netherlands; e Institute for Complex Molecular Systems, Eindhoven University of Technology, Eindhoven, the Netherlands; f Department of Industrial Engineering and Innovation Sciences, Eindhoven University of Technology, Eindhoven, the Netherlands; g Netherlands Heart Institute, Utrecht, the Netherlands

**Keywords:** Out-of-hospital cardiac arrest, Sudden cardiac death, Telemedicine, User-computer interface

## Abstract

**Aim:**

Out-of-hospital cardiac arrest (OHCA) is a leading cause of mortality, and rapid treatment is life-saving. Early detection is crucial to promptly start the chain of survival, leading to increasing interest in smartwatch-based OHCA detection. Introducing a grace period, during which the wearer can cancel a false alarm before emergency medical services (EMS) are notified, may improve system reliability. This study evaluates how this grace period affects false alarm rates.

**Methods:**

In this study, 26 participants wore smartwatches that produced auditory, tactile or audiotactile alarms at random times during daytime, while instructed to cancel these alarms as quickly as possible. Response times were registered by the smartwatch, alongside demographic and time-of-day data. Bayesian time-to-event analysis assessed the effects of alarm type, time of day, and demographic variables.

**Results:**

(Audio)tactile alarms significantly shortened response times compared to auditory-only alarms (HR 0.475, 95% CI: 0.38–0.59). Grace periods of 10 and 20 s would result in 98.3% (95% CI: 97.1–99.0%) and 99.6% (95% CI: 99.2–99.9%) of the (audio)tactile alarms being canceled, respectively. No clear evidence was found for meaningful effects of time of day, age or sex.

**Conclusion:**

The findings in this study suggest that the application of a grace period to smartwatch-based OHCA detection systems may potentially reduce false alarms reaching EMS with only minor delays. Further research is warranted in a larger implementation set-up.

## Introduction

Cardiac arrest is a significant global health issue, causing numerous deaths worldwide.^1^, ^2^, ^3^, ^4^, ^5^, ^6^ Sudden out-of-hospital cardiac arrest (OHCA) is among the leading causes of mortality in Europe.^5^, ^7^ There are approximately 408,000 cases occurring annually.^1^ In the European registry of cardiac arrest (EuReCa) studies, the overall survival rate of the OHCA cases where cardiopulmonary resuscitation (CPR) was attempted was 7.5%.^4^

The time to first shock is a critical determinant of survival rate in OHCA cases.^7^, ^8^ One study indicates that every additional minute between the emergency call and the delivery of the first shock decreases the probability of survival up to hospital discharge by 6%.^7^ As victims of unwitnessed OHCA often experience delayed treatment, they face much lower chances of survival. While advances in the chain of survival have contributed to increased survival rates following OHCA,^3^, ^9^, ^10^, ^11^, ^12^, ^13^ a critical barrier to further improvement remains the need for early detection of OHCA in unwitnessed cases, in order to promptly initiate the chain of survival.^14^

**Fig. 1:**
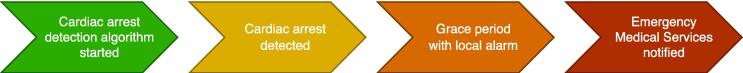
**Flowchart of OHCA detection to EMS notification**.

The BECA (BEating Cardiac Arrest) project is developing a smartwatch capable of autonomously detecting OHCA and alerting emergency medical services (EMS) and first responders, essentially functioning as a witness.^14^ To prevent the device from sending false alarms to the EMS, a grace period will be introduced: a time window in which the wearer of the watch can cancel a local alarm before automatic EMS notification (see Fig. 1). The false alarm rate, expressed in false alarms per unit time, will therefore depend on the percentage of wearers able to respond to a local alarm before the end of the grace period.

A longer grace period generally leads to a lower false alarm rate, as wearers have more time to cancel the alarm. However, this also leads to a delay in activating the chain of survival. Given this trade-off, the duration of the grace period forms an important factor in the concept of smartwatch-based OHCA detection systems. In this study, we simulate smartwatch OHCA alarms and evaluate the effects of the grace period on the false alarm rate, including the effects of grace period duration, the type of local alarm being used, time of day, and demographic background of the wearer.

## Methods

### Data acquisition

In this behavioral study, data were collected from participants of different sexes, divided in a ‘young’ (20–25 years) and ‘old’ (56–64 years) group, allowing us to analyze the effects of sex and age. While cardiac arrest is more prevalent in older people, young people can also suffer from cardiac arrest, and were therefore considered in this study as well.^1^

All participants were instructed to wear a LilyGo T-Watch 2020V3 programmable smartwatch for one day between 12 PM and 10 PM and to cancel any alarm produced by the watch as quickly as possible by tapping its screen. The smartwatch recorded the response time from alarm onset to cancelation. These response times formed the basis for analyzing the effects of grace period duration on the false alarm rate, as the user response time determines whether the user will be able to cancel a false alarm within the grace period. The exact times of turning the watches on/off were not registered.

In real-world application, failing to cancel a false alarm results in unnecessarily alerting EMS, creating a sense of urgency in users to cancel an alarm in time. To replicate this sense of urgency, we informed participants about the real-world consequences of false alarms, and included extra monetary compensation (€5) for the two fastest participants in each age group on top of base compensation (€12-€15). As a complete focus on canceling the alarm as fast as possible may lead to dangerous situations (e.g., while driving a car), we instructed the participants to prioritize safety over response time. Furthermore, to avoid alarms to continue indefinitely, an uncancelled alarm ceased automatically after 60 s and was marked as censored.

During each trial, the watch produced 10–19 alarms on a predefined schedule unknown to the participant. At the scheduled time of an alarm, the watch checked for a window of 10 s of motionlessness using its accelerometer before activating the alarm, as motionlessness was expected to be an indicator considered by OHCA detection algorithms. This expectation was later confirmed by the DETECT-1 study, which shared this expectation,^15^ and the Google Research’s loss of pulse study, which implemented this indicator in their algorithm.^16^ If no window of motionlessness could be detected within 10 min, the scheduled alarm was raised, but its corresponding response time was excluded from this analysis. This mechanism was implemented to prevent participants from thinking the programmable watch was malfunctioning and to potentially gain insights into the response times for these cases.

Three alarm types were randomly scheduled:

• *Auditory*: four beeps (at 2050 and 4100 Hz) of 60 ms with pauses of 60 ms, followed by 580 ms of silence.
• *Tactile*: three vibration pulses (at 60 Hz) of 500 ms with pauses of 400 ms, followed by an 800 ms pulse.
• *Audiotactile*: combination of auditory and tactile alarms.

During all alarms, the display blinked (500 ms on, 500 ms off).

### Statistical analysis

Given the nature of the data, time-to-event analysis was applied to model the response times. Response times were considered to be right-censored when no response occurred within the observation window. A shared frailty model with a log-logistic baseline hazard function and gamma distributed frailty with unit mean was used to analyze these response times.^17^ This model assumes proportional hazards, with the frailty term acting multiplicatively on the individual hazard functions. A frailty component was included because multiple response times were recorded for each participant, potentially inducing within-participant correlation. The log-logistic baseline hazard was chosen because it provides flexibility in modeling time-to-event distributions that are skewed, with many events occurring early and fewer occurring later.

We drew inference using a Bayesian approach.^18^ We included the variables age as a binary variable divided between the younger and older group, sex, time of day as a binary variable divided in afternoon and evening to the Bayesian frailty model. We also included the alarm type, where we added the binary variables for auditory and tactile alarms and have audiotactile alarms as a reference. To conduct variable selection, we investigated the 95% credible intervals (CIs) of the hazard ratios and retained the variables whose intervals excluded 1.

The parameters of the log-logistic baseline hazard function and the frailty distribution, which were modeled on the log scale, as well as the regression coefficients for the included variables, were each assigned independent standard normal priors to provide weakly informative regularization on an unconstrained scale.^18^ A total of four Markov chains were run with 2000 iterations each of which 1000 warm-up and 1000 sampling iterations. The convergence was assessed using the potential scale reduction statistic, the Rhat, and visual inspection of trace plots. To assess the predictive performance of the final model, we assessed both calibration and discrimination. Discrimination was quantified using the concordance-index (C-index), which measures the model’s ability to correctly rank participants by risk. Calibration and overall accuracy of predicted cumulative incidence rates were assessed using the time-dependent Brier score.

For the analysis, we used R (version 4.2.1, “Funny-Looking Kid”) with Stan via the *rstan* package.^19^, ^20^ The auditory and tactile alarms were added to the model with the audiotactile alarms as a reference. The hazard ratio (HR) and time-to-event probabilities are reported. Continuous variables were expressed as mean (±standard deviation) or median (interquartile range, IQR), while categorical variables are shown as counts (frequency) where appropriate. Kaplan-Meier cumulative incidence plots with its 95% confidence intervals were generated using the *lifelines* library in Python.^21^

## Results

### Descriptive analysis

Data were collected from 26 participants, where one participant was excluded from analysis because of missing demographic data. We analyzed 25 participants: a younger group of 14 participants with a mean age of 21.9 years (SD: 1.4), of which 10 (71%) were male, and an older group of 11 participants with a mean age of 59.7 years (SD: 2.5), of which 5 (45%) were male. The watches produced 136 auditory (33%), 141 tactile (34%) and 139 audiotactile (33%) alarms. In total, 229 (55%) alarms went off in the afternoon and 187 (45%) in the evening (afternoon 12PM-5PM, evening 5PM-10PM). A total of 6 alarms were censored, as they were not canceled within 60 s. Due to the low number of censored alarms, no conclusions could be drawn on reasons why alarms could not be canceled. An additional 6 alarms were excluded from analysis, as no period of motionlessness could be found by the watch.

**Fig. 2:**
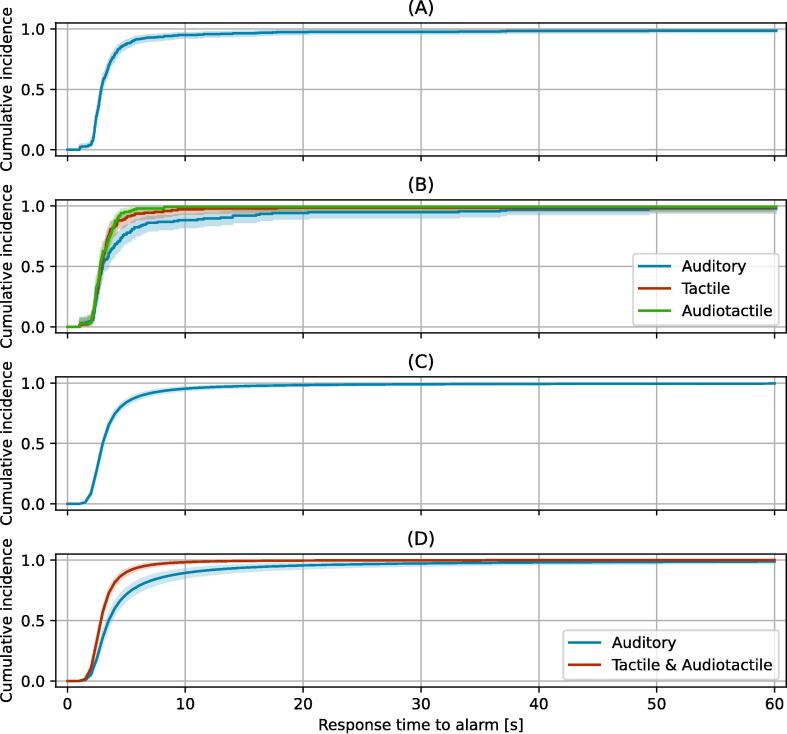
**(A) Non-parametric Kaplan-Meier estimates of the cumulative incidence of response times with their 95% confidence interval for all alarms combined. (B) Non-parametric Kaplan-Meier estimates of the cumulative incidences of response times with their 95% confidence interval, separated by alarm type. (C) Posterior mean cumulative incidence curves with 95% credible intervals obtained from the Bayesian model for all alarms combined. (D) Posterior mean cumulative incidence curves with 95% credible intervals obtained from the Bayesian model, separated by Auditory alarms and the combination of Tactile and Audiotactile alarms**.

To get an impression of the response time data, we plotted Kaplan-Meier cumulative incidence curves for all alarms in Fig. 2A and stratified per alarm in Fig. 2B. The curves show that the cumulative incidence rises steeply in the first few seconds, indicating that most participants responded quickly after the alarm was raised. While the response times for different alarms show the same sharp rise at the beginning, the cumulative incidence for the auditory alarm is lower than those of the tactile and audiotactile alarms. The median response time for all alarms is 2.88 s (IQR: 1.35). The median response is 2.99 s (IQR: 2.19) for auditory alarms, 2.86 s (IQR: 1.07) for tactile alarms, and 2.76 s (IQR: 1.22) for audiotactile alarms. In Supplementary Table 1 we report the number of alarms at risk, censored and the number of responses at 5-s intervals for each alarm type. This gives the counts that correspond to Fig. 2A and B.

### Bayesian time-to-event analysis

The credible intervals of the hazard ratios of auditory vs. tactile alarms (HR: 0.47, 95% CI: 0.36–0.61) and audiotactile vs. auditory alarms (HR: 0.50, 95% CI: 0.39–0.65) excludes 1, indicating an effect of longer response times when using auditory alarms. The variable tactile vs. audiotactile alarms (HR: 1.07, 95% CI: 0.83–1.34) includes 1 in its credible interval, suggesting no clear evidence of an effect. The other variables, afternoon vs. evening alarms (HR: 0.91, 95% CI: 0.74–1.12), ‘young’ vs. ‘old’ participants (HR: 1.36, 95% CI: 0.70–2.49) and male vs. female participants (HR: 0.59, 95% CI: 0.30–1.06), all include 1 in their credible intervals, suggesting no clear evidence of an effect.

This means that only the auditory alarm has an effect on the response time for the participants. Hence, we consider audiotactile and tactile alarms as one group. This gives a hazard ratio of 0.48 (95% CI: 0.38–0.59), indicating an effect. In Fig. 2C, we show the cumulative incidence curve for all alarms, and in Fig. 2D, we show the cumulative incidence curves for the tactile & audiotactile alarm group and the auditory alarms separately.

At a grace period of, for example, 10 s, tactile and audiotactile alarms resulted in 98.3% of the alarms being canceled (95% CI: 97.1–99.0%), while auditory alarms resulted in 89.4% cancelation (95% CI: 85.2–92.6%). For a grace period of 20 s, these numbers change to 99.6% (95% CI: 99.2–99.9%) and 95.5% (95% CI: 92.8–97.4%) respectively. We will consider the effects of these example grace period durations in the Discussion section. Durations longer than 20 s do not substantially improve the cumulative incidence, and are not considered further. In Supplementary Table 2, we show the cumulative incidences between 0 and 60 s at 5-s intervals.

In Supplementary Table 3 we show the values for the Brier score and C-index across time. *Here*, it can be seen that there is no indication that there is a bad predictive performance. The traceplots of the parameters that were estimated using the Bayesian time-to-event model are depicted in Supplementary Fig. 1. The traceplots show good mixing across all four chains used, with no signs of drift, divergence or non-stationarity. Moreover, the values for Rhat are either 1.00 or 1.01 for all parameters, meaning that there is no evidence of lack of convergence. Supplementary Table 4 shows the estimated hazard ratios under different choices of prior distributions. For different choices of prior distributions, the values of the hazard ratios remain very close to each other.

## Discussion

Our results show that almost all smartwatch alarms could be canceled within 10 to 20 s. After such a period, little gain in the number of canceled alarms can be expected. We can also conclude that alarms containing a tactile component yield faster response times than alarms with solely an audio component. Remarkably, demographic factors such as age and sex had no significant effect on response times, emphasizing the robustness of (audio)tactile alarms across diverse populations.

Given these results, implementing a grace period of 10–20 s with an (audio)tactile alarm would yield considerable advantages in terms of false alarm reduction. A recent study by the HEART-SAFE consortium reported on an OHCA detection method that yields a sensitivity of 90.3% and an hour-level specificity of 94.1%, corresponding to 511 false alarms per user-year.^22^ Similarly, the DETECT project reported a sensitivity of 98% and a minute-level specificity of 99.9% based on their DETECT-1 study, corresponding to 526 false alarms per user-year.^15^ When such technologies would be rolled out en masse, it would produce an unreasonable amount of false alarms, which discourages stakeholders to adopt smartwatch-based OHCA detection technology. After all, EMS would be overburdened by these false alarms, citizen first responders could be demotivated in participating, and users could be discouraged from wearing their watches.

However, the addition of a 10-s grace period would reduce the number of false alarms per user-year to 8.69 and 8.94, and a 20-s grace period would result in 2.04 and 2.10 false alarms per user-year, respectively, resulting in a massive reduction in false alarm rates. While we suspect that 2 false alarms per user-year is still too many for practical implementation of such OHCA detection methods, the addition of the grace period already contributes a lot to the practicality of implementation.

Another major advantage of implementing a grace period is the overall improvement of diagnostic performance, which benefits methods that suffer from low sensitivity. Google Research’s loss of pulse study reported statistics corresponding to a day-level specificity of 99.6% of the detection algorithm, which equals 1.43 false alarms per user-year, and a relatively low sensitivity of 67.23%.^16^ While the reported specificity should be higher for practical implementation, significant improvements in sensitivity can be gained by trading a high specificity for an increase in sensitivity. This can be achieved by lowering the diagnostic threshold for OHCA detection. After that, the addition of a grace period could offset the reduced specificity.

The addition of a grace period also yields a major disadvantage in terms of OHCA survival rate. Given that every minute of delay reduces the likelihood of survival to hospital discharge 6%^7^, introducing or prolonging the grace period by 10 or 20 s could significantly reduce survival chances. To provide a coarse estimate based on the mentioned statistics, every additional 10 s of grace period would lead to a $0.94^{10/60}\times 100\%=10.\%$ reduction in survival to discharge rate. Comparing our recommended grace period of 10–20 s with Google Research’s proposed grace period of 35 s, their proposed grace period would result in a 1.5% to 2.5% decrease in survival to discharge rate. Given that this additional 15–25 s would not yield significantly more false alarm cancelations, we would recommend to shorten their grace period significantly.

Considering these advantages and disadvantages, it becomes clear that (1) the implementation of a grace period has significant positive effects on the overall practicality of implementation, and (2) that a trade-off between false alarm rate and OHCA survival rate is clearly present. Both are reasons to consider the duration of the grace period and the type of alarm that is produced during this period.

Up to this point, only Google Research has considered the effect of a grace period in their work. They reported that for 31 false alarms, a tactile notification leads to 90% of the alarms being canceled within 15 s, and a follow-up audiotactile notification leads to 97% of the alarms being canceled within an additional 20 s.^16^ These results are different from our results, as we showed that 99.9% (95% CI: 99.6–100%) of the alarms could have been canceled within 35 s. However, as admitted by the authors of Google Research, their numbers are probably conservative underestimates. Furthermore, the low number of actual alarms produced and the fixed grace period duration do not show as thorough of an analysis as done in this work.

### Limitations

While this study provides valuable insights into the effects of a grace period, there are several important limitations to consider. First, the auditory alarms used were not particularly loud, which may have contributed to the relatively longer response times. In practice, one could consider increasing the volume of the sound to capture attention more quickly. However, we must also consider compliance, as loud false alarms may discourage owners from wearing such watches or activate the OHCA detection functionality. In that sense, tactile-only alarms should be considered, as these may lead to stronger compliance compared to auditory alarms, while requiring a similar grace period duration.

Additionally, to mimic a user’s sense of urgency to cancel a false alarm before alerting the EMS, we explained to the participants what the real-world consequences of false alarms would be and provided monetary rewards for faster response times. However, these incentives may not have fully or accurately replicated this sense of urgency and, therefore, may have biased the response times.

Moreover, the older group within the study population had a mean age of 59.7 years old, which is notably younger than the typical cardiac arrest population. For instance, studies reported a mean age of 67.6 years for EMS-witnessed cases and 66.3 years for all treated OHCA cases.^23^, ^24^ Although this study concluded that there is no clear evidence that older individuals respond differently than younger individuals, there is also insufficient evidence to rule out no effect. Therefore, further research on the effects of age would be recommended.

Furthermore, for practical and ethical reasons, we decided not to test alarms during nighttime. Therefore, the presented results should always be interpreted in a daytime context. During sleep, responses may be delayed, requiring a longer grace period. With the current developments in smartwatch-based sleep staging, an adaptive grace period could be introduced, which would allow for separate daytime and nighttime grace period settings.^25^ We strongly recommend that follow-up studies include nighttime studies.

Lastly, the study was conducted in a setting where participants were aware that alarms would be produced during the day, which may have affected their cognitive focus and readiness to respond. This awareness might not fully represent real-world situations where alarms can occur unexpectedly, sometimes amidst distractions or environmental noise. Furthermore, as the watches used in this study had the sole functionality of producing alarms, the effects of other smartwatch functions were not considered, e.g., notification fatigue due to incoming text messages.

## Conclusion

As interest in smartwatch-based OHCA detection systems grows, understanding the role of the grace period between algorithm detection and EMS notification is crucial. While a longer grace period may reduce false alarms, it also delays intervention and lowers survival rates. In this behavioral study, testing the response times to smartwatch alarms with a simulated sense of urgency revealed that grace period durations of 10 and 20 s would result in cancelation of 98.3% (95% CI: 97.1–99.0%) and 99.6% (95% CI: 99.2–99.9%) of false alarms during daytime, respectively. Alarms with a tactile component were associated with shorter response times compared with auditory alarms, while the data did not provide sufficient evidence to identify meaningful effects of factors such as age, sex, or time of day. These findings suggest that applying a grace period to existing OHCA detection methods may potentially reduce false alarms reaching EMS with only minor delays, warranting further investigation in a larger implementation set-up.

## Ethics declaration

This study was performed in compliance with relevant laws and institutional guidelines, including the Declaration of Helsinki. Approval of the study was obtained from the Ethical Review Board of the Human Technology Interaction group at Eindhoven University of Technology (ref. 1906). The privacy rights of the participants have been observed, and written informed consent was obtained from each participant.

## CRediT authorship contribution statement

**Roelof G. Hup:** Writing – original draft, Visualization, Validation, Supervision, Software, Resources, Methodology, Formal analysis, Data curation, Conceptualization. **Chaimae Bouchnaf:** Writing – original draft, Validation, Methodology, Formal analysis, Data curation, Conceptualization. **Myrthe A. Plaisier:** Writing – review & editing, Supervision, Resources, Methodology, Formal analysis, Conceptualization. **Fatuma M.A. Omar:** Writing – original draft, Visualization, Software, Methodology, Formal analysis. **Tobias A. Machiavello:** Writing – review & editing, Validation, Software, Methodology, Investigation, Formal analysis, Data curation, Conceptualization. **Sophie L.M. van Spreuwel:** Writing – review & editing, Validation, Software, Methodology, Investigation, Formal analysis, Data curation, Conceptualization. **Hanno L. Tan:** Writing – review & editing, Supervision, Funding acquisition, Conceptualization. **Xi Long:** Writing – review & editing, Supervision. **Rik Vullings:** Writing – review & editing, Supervision, Methodology, Funding acquisition, Conceptualization.

## Funding

The BECA project is financed by the PPP Allowance made available by Top Sector Life Sciences & Health to the Dutch Heart Foundation to stimulate public–private partnerships, grant number 01-003-2021-B005, and by Philips Electronics Nederland B.V.

## Declaration of competing interest

The authors declare the following financial interests/personal relationships which may be considered as potential competing interests: The BECA project is financed by the PPP Allowance made available by Top Sector Life Sciences & Health to the Dutch Heart Foundation to stimulate public–private partnerships, grant number 01-003-2021-B005, and by Philips Electronics Nederland B.V. R. Vullings has a consultancy position at Philips, the Netherlands.

## Data Availability

Anonymized data from this study have been deposited at the 4TU.ResearchData repository under DOI https://doi.org/10.4121/e1a2752f-7b98-45e2-bb61-7b1d3eec0b0c. The code for statistical analysis is available at https://github.com/fatuma-omar/Project-Grace-Period-BECA.
