# Supplementary figures and images for: Effect of a grace period on false alarm rates of smartwatch-based out-of-hospital cardiac arrest detection systems: a pilot study
Source: Resusc Plus. 2026 Jan 5;28:101215. doi: 10.1016/j.resplu.2025.101215 (PMC12835406; doi:10.1016/j.resplu.2025.101215)

Supplementary Figure 1: Traceplots of parameters

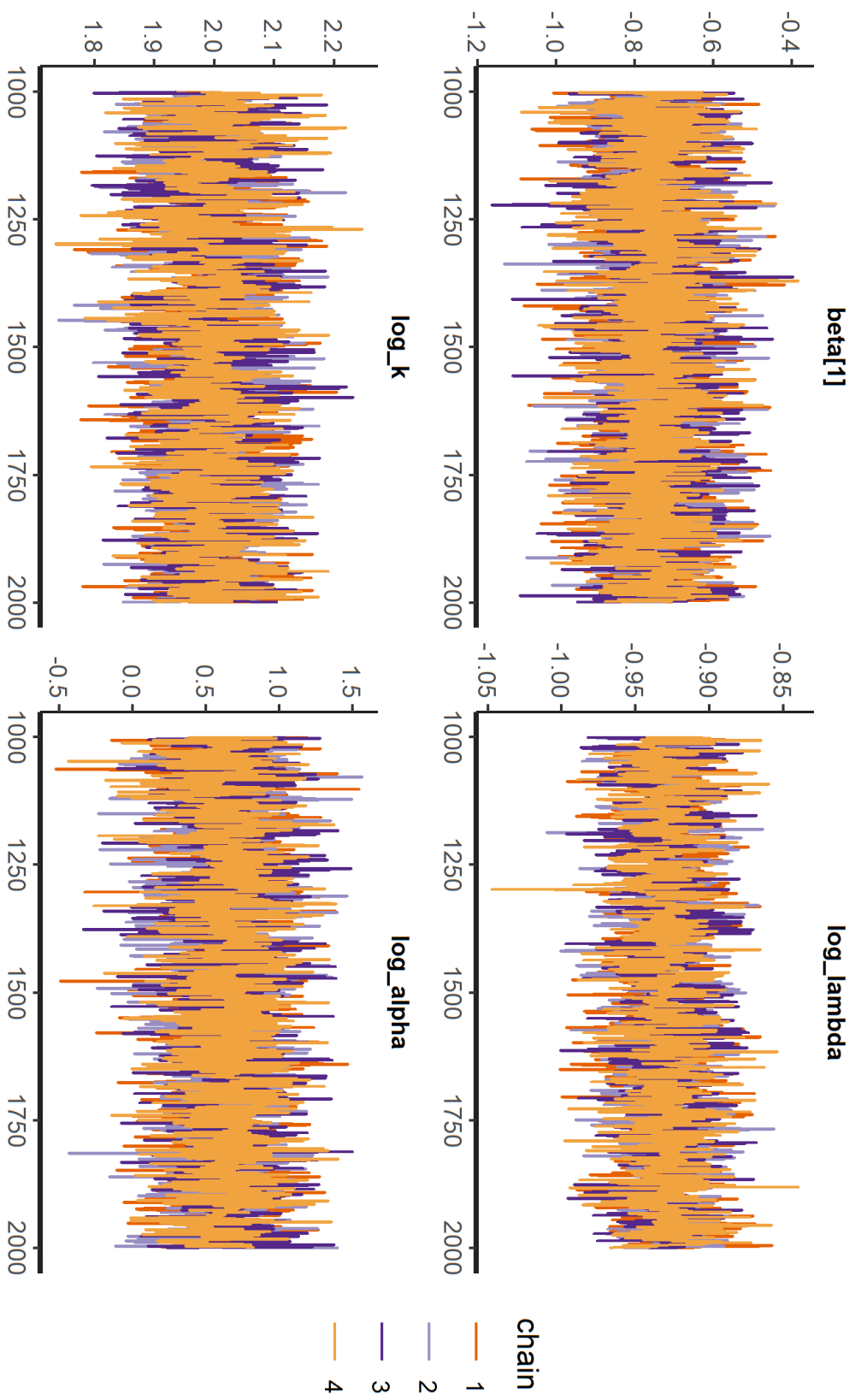

Supplement: Supplementary Fig. 1 [file mmc1.pdf]
