# Supplementary material for: Effect of a grace period on false alarm rates of smartwatch-based out-of-hospital cardiac arrest detection systems: a pilot study
Source: Resusc Plus. 2026 Jan 5;28:101215. doi: 10.1016/j.resplu.2025.101215 (PMC12835406; doi:10.1016/j.resplu.2025.101215)
Supplement: Supplementary Table 1 [file mmc2.pdf]

Supplementary Table 1: Event tables

Response time to alarm [s]	All alarms			Auditory			Tactile			Audiotactile		
	O[1]	C[2]	N risk[3]	O[1]	C[2]	N risk[3]	O[1]	C[2]	N risk[3]	O[1]	C [2]	N risk[3]
0	0	0	416	0	0	136	0	0	141	0	0	139
5	363	0	53	104	0	32	127	0	14	132	0	7
10	395	0	21	120	0	16	137	0	4	138	0	1
15	401	0	15	125	0	11	138	0	3	138	0	1
20	405	0	11	128	0	8	139	0	2	138	0	1
25	406	0	10	129	0	7	139	0	2	138	0	1
30	406	0	10	129	0	7	139	0	2	138	0	1
35	407	0	9	130	0	6	139	0	2	138	0	1
40	409	0	7	132	0	4	139	0	2	138	0	1
45	409	0	7	132	0	4	139	0	2	138	0	1
50	410	0	6	133	0	3	139	0	2	138	0	1
55	410	0	6	133	0	3	139	0	2	138	0	1
60	410	6	0	133	3	0	139	2	0	138	1	0

[1] Observed events [2] Censored events [3] Events at risk
