# Supplementary material for: Effect of a grace period on false alarm rates of smartwatch-based out-of-hospital cardiac arrest detection systems: a pilot study
Source: Resusc Plus. 2026 Jan 5;28:101215. doi: 10.1016/j.resplu.2025.101215 (PMC12835406; doi:10.1016/j.resplu.2025.101215)
Supplement: Supplementary Table 2 [file mmc3.pdf]

# Supplementary Table 2: Modeled cumulative incidences

| Response time to alarm [s] | All alarms | | | Auditory | | | Tactile & Audiotactile | | |
|---|---|---|---|---|---|---|---|---|---|
| | CIF[1] | LCL[2] | UCL[3] | CIF[1] | LCL[2] | UCL[3] | CIF[1] | LCL[2] | UCL[3] |
| 0 | 0.000 | 0.000 | 0.000 | 0.000 | 0.000 | 0.000 | 0.000 | 0.000 | 0.000 |
| 5 | 0.841 | 0.814 | 0.864 | 0.718 | 0.663 | 0.768 | 0.901 | 0.872 | 0.924 |
| 10 | 0.953 | 0.937 | 0.966 | 0.894 | 0.852 | 0.926 | 0.983 | 0.971 | 0.990 |
| 15 | 0.974 | 0.962 | 0.983 | 0.937 | 0.904 | 0.960 | 0.993 | 0.986 | 0.997 |
| 20 | 0.983 | 0.973 | 0.990 | 0.955 | 0.928 | 0.974 | 0.996 | 0.992 | 0.999 |
| 25 | 0.987 | 0.979 | 0.993 | 0.966 | 0.942 | 0.981 | 0.998 | 0.994 | 0.999 |
| 30 | 0.990 | 0.982 | 0.995 | 0.972 | 0.952 | 0.985 | 0.998 | 0.996 | 0.999 |
| 35 | 0.992 | 0.985 | 0.996 | 0.977 | 0.958 | 0.988 | 0.999 | 0.996 | 1.000 |
| 40 | 0.993 | 0.987 | 0.996 | 0.980 | 0.963 | 0.990 | 0.999 | 0.997 | 1.000 |
| 45 | 0.994 | 0.988 | 0.997 | 0.983 | 0.967 | 0.992 | 0.999 | 0.998 | 1.000 |
| 50 | 0.994 | 0.989 | 0.997 | 0.984 | 0.970 | 0.993 | 0.999 | 0.998 | 1.000 |
| 55 | 0.995 | 0.990 | 0.998 | 0.986 | 0.972 | 0.994 | 1.000 | 0.998 | 1.000 |
| 60 | 0.996 | 0.991 | 0.998 | 0.987 | 0.974 | 0.994 | 1.000 | 0.998 | 1.000 |

[1] Cumulative incidence [2] Lower 95% credible interval limit [3] Upper 95% credible interval limit
