# Supplementary material for: Effect of a grace period on false alarm rates of smartwatch-based out-of-hospital cardiac arrest detection systems: a pilot study
Source: Resusc Plus. 2026 Jan 5;28:101215. doi: 10.1016/j.resplu.2025.101215 (PMC12835406; doi:10.1016/j.resplu.2025.101215)
Supplement: Supplementary Table 3 [file mmc4.pdf]

Supplementary Table 3: Brier score and concordance index (C-index)

Response time to alarm [s]	Posterior mean Brier score			Posterior mean C-index		
	value	LCL[1]	UCL[2]	value	LCL[1]	UCL[2]
0	0.000	0.000	0.000	0.000	0.000	0.000
5	0.211	0.200	0.223	0.721	0.686	0.754
10	0.106	0.101	0.112	0.753	0.728	0.773
15	0.047	0.045	0.051	0.750	0.703	0.790
20	0.035	0.033	0.036	0.760	0.707	0.805
25	0.026	0.025	0.027	0.742	0.681	0.797
30	0.024	0.023	0.025	0.738	0.681	0.797
35	0.023	0.023	0.024	0.738	0.675	0.797
40	0.021	0.021	0.022	0.753	0.688	0.811
45	0.017	0.016	0.017	0.716	0.643	0.783
50	0.017	0.016	0.017	0.716	0.643	0.783
55	0.014	0.014	0.015	0.742	0.670	0.811
60	0.014	0.014	0.015	0.742	0.670	0.811

[1] Lower 95% credible interval limit [2] Upper 95% credible interval limit
