# Supplementary material for: Effect of a grace period on false alarm rates of smartwatch-based out-of-hospital cardiac arrest detection systems: a pilot study
Source: Resusc Plus. 2026 Jan 5;28:101215. doi: 10.1016/j.resplu.2025.101215 (PMC12835406; doi:10.1016/j.resplu.2025.101215)
Supplement: Supplementary Table 4 [file mmc5.pdf]

Supplementary Table 4: Sensitivity analysis of hazard ratio with different choices of prior distributions

Prior distribution	Posterior mean hazard ratio (95% Credible Interval)			
	Normal(0, 1)	Normal(0, 0.5)	Student-t(1, 0, 5)	Student-t(3, 0, 0.5)
Audiotactile vs. tactile	1.07 (0.83 – 1.34)	1.09 (0.86 – 1.38)	1.07 (0.83 – 1.36)	1.08 (0.85 – 1.35)
Auditory vs. tactile	**0.50 (0.39 – 0.65)**	**0.52 (0.41 – 0.66)**	**0.50 (0.38 – 0.64)**	**0.52 (0.40 – 0.66)**
Audiotactile vs. auditory	**0.47 (0.36 – 0.61)**	**0.48 (0.37 – 0.61)**	**0.47 (0.36 – 0.60)**	**0.48 (0.37 – 0.62)**
Young vs. old	1.36 (0.70 – 2.49)	1.16 (0.64 – 1.90)	1.46 (0.72 – 2.67)	1.17 (0.65 – 2.00)
Afternoon vs. evening	0.91 (0.74 – 1.12)	0.92 (0.74 – 1.12)	0.91 (0.74 – 1.11)	0.92 (0.75 – 1.11)
Male vs. female	0.59 (0.30 – 1.06)	0.68 (0.39 – 1.16)	0.56 (0.28 – 1.01)	0.68 (0.37 – 1.17)
